# Assessment of Simulated Surveillance Testing and Quarantine in a SARS-CoV-2–Vaccinated Population of Students on a University Campus

**DOI:** 10.1001/jamahealthforum.2021.3035

**Published:** 2021-10-01

**Authors:** Francis C. Motta, Kevin A. McGoff, Anastasia Deckard, Cameron R. Wolfe, Mattia Bonsignori, M. Anthony Moody, Kyle Cavanaugh, Thomas N. Denny, John Harer, Steven B. Haase

**Affiliations:** 1 Department of Mathematical Sciences, Florida Atlantic University, Boca Raton; 2 Department of Mathematics and Statistics, University of North Carolina, Charlotte; 3 Office of Information Technology, Duke University, Durham, North Carolina; 4 Department of Medicine, Duke University School of Medicine, Durham, North Carolina; 5 Laboratory of Infectious Diseases, National Institute of Allergy and Infectious Diseases, National Institutes of Health, Bethesda, Maryland; 6 Department of Medicine, Duke Human Vaccine Institute, Duke University School of Medicine, Durham, North Carolina; 7 Department of Pediatrics and Duke Human Vaccine Institute, Duke University School of Medicine, Durham, North Carolina; 8 Office of Vice President of Administration, Department of Family Medicine, Duke University School of Medicine, Durham, North Carolina; 9 Department of Mathematics, Duke University, Durham, North Carolina; 10 Departments of Biology and Medicine, Duke University, Durham, North Carolina

## Abstract

**Importance:**

The importance of surveillance testing and quarantine on university campuses to limit SARS-CoV-2 transmission needs to be reevaluated in the context of a complex and rapidly changing environment that includes vaccines, variants, and waning immunity. Also, recent US Centers for Disease Control and Prevention guidelines suggest that vaccinated students do not need to participate in surveillance testing.

**Objective:**

To evaluate the use of surveillance testing and quarantine in a fully vaccinated student population for whom vaccine effectiveness may be affected by the type of vaccination, presence of variants, and loss of vaccine-induced or natural immunity over time.

**Design, Setting, and Participants:**

In this simulation study, an agent-based Susceptible, Exposed, Infected, Recovered model was developed with some parameters estimated using data from the 2020 to 2021 academic year at Duke University (Durham, North Carolina) that described a simulated population of 5000 undergraduate students residing on campus in residential dormitories. This study assumed that 100% of residential undergraduates are vaccinated. Under varying levels of vaccine effectiveness (90%, 75%, and 50%), the reductions in the numbers of positive cases under various mitigation strategies that involved surveillance testing and quarantine were estimated.

**Main Outcomes and Measures:**

The percentage of students infected with SARS-CoV-2 each day for the course of the semester (100 days) and the total number of isolated or quarantined students were estimated.

**Results:**

A total of 5000 undergraduates were simulated in the study. In simulations with 90% vaccine effectiveness, weekly surveillance testing was associated with only marginally reduced viral transmission. At 50% to 75% effectiveness, surveillance testing was estimated to reduce the number of infections by as much as 93.6%. A 10-day quarantine protocol for exposures was associated with only modest reduction in infections until vaccine effectiveness dropped to 50%. Increased testing of reported contacts was estimated to be at least as effective as quarantine at limiting infections.

**Conclusions and Relevance:**

In this simulated modeling study of infection dynamics on a college campus where 100% of the student body is vaccinated, weekly surveillance testing was associated with a substantial reduction of campus infections with even a modest loss of vaccine effectiveness. Model simulations also suggested that an increased testing cadence can be as effective as a 10-day quarantine period at limiting infections. Together, these findings provide a potential foundation for universities to design appropriate mitigation protocols for the 2021 to 2022 academic year.

## Key Points

**Question:**

Are surveillance testing and quarantine still important for limiting virus transmission on college campuses where the student population is completely vaccinated against SARS-CoV-2?

**Findings:**

In this decision analytical model study of 5000 simulated undergraduates, if 100% were vaccinated with 90% vaccine effectiveness, surveillance testing and quarantine were not associated with a substantial reduction in infections. However, if vaccine effectiveness was reduced to 75%, weekly surveillance testing was associated with a substantial reduction in the number of infections; at 50% vaccine effectiveness, surveillance testing and quarantine were associated with a marked reduction in the estimated number of infections.

**Meaning:**

The study results suggest that surveillance testing and isolation of positive cases may remain important mitigation strategies on university campuses with a vaccinated student body with only modest erosion of vaccine effectiveness, while quarantining contacts offers limited added benefit over surveillance testing alone and may be effectively replaced by an increased testing cadence of reported contacts.

## Teaser

This decision analytical model study examines the use of surveillance testing and quarantine in a fully COVID-19–vaccinated university student population.

## Introduction

Surveillance testing and quarantine were 2 approaches used successfully by universities to limit the spread of SARS-CoV-2 during the 2020 to 2021 academic year.^1^ In the coming year, many universities are looking to vaccinations as the foundation of their COVID-19 mitigation programs, with some requiring a SARS-CoV-2 vaccination to live and attend classes on campus. If 100% of the student population is vaccinated, will surveillance testing and quarantine still be important for preventing viral transmission among the student body?

If all students were vaccinated and the vaccinations were 100% effective, then surveillance testing and quarantine would be unnecessary. However, the BioNTech 162b2 and messenger RNA-1273 vaccines exhibit approximately 90% effectiveness against infection,^2,3,4^ and the Ad26.COV2.S vaccine is approximately 66% effective.^5^ Moreover, some genetic variants of SARS-CoV-2 (eg, B.1.351 [β] and P1 [γ]) appear to have some resistance to vaccine-induced neutralizing antibodies.^6,7,8,9^ Thus, it is possible that the effectiveness of vaccines may be further eroded by the presence of known and future viral variants.^10^ Finally, the durability of vaccine-induced immunity is still unknown, and immunity may wane over time.^11,12^ These factors are likely to contribute to an uncertain and continually changing environment of immunity. In such an environment, it is conceivable that surveillance testing and quarantine might remain important COVID-19 mitigation strategies on college campuses, in contrast to recent US Centers for Disease Control and Prevention (CDC) guidelines.^13^

Mathematical models have been useful tools for exploring SARS-CoV-2 infection dynamics and the effects of various mitigations on university campuses.^14,15,16,17^ This study uses simulated infection dynamics of an agent-based, modified Susceptible, Exposed, Infected, Recovered (SEIR) model to investigate the effect of surveillance testing and quarantine in an environment where 100% of the student population is vaccinated, but where vaccine effectiveness may be reduced by variants or by waning immunity. The added reduction in viral transmission because of quarantining reported contacts or increasing surveillance testing frequency over weekly surveillance testing alone was also estimated.

## Methods

An agent-based model with SEIR disease dynamics was developed to simulate SARS-CoV-2 spread among an on-campus population of 5000 homogeneously mixing agents. The model also incorporated an off-campus population, which was modeled as a reservoir with a static prevalence of infection. Each day each susceptible agent could randomly become exposed through a fixed number of interactions with the on-campus and the off-campus populations. Each agent's probability of exposure depended on the prevalence of infection in the (nonisolated and nonquarantined) populations with which it was interacting and on a fixed probability that an interaction would lead to an exposure. Because the probability that an interaction will result in an exposure is highly context-specific and difficult to estimate,^18,19,20^ the transmission probability because of an interaction was arbitrarily fixed; thus, it implicitly defined the type of interactions being modeled via this probability. Reasonable numbers of such interactions were estimated by simultaneously searching for the number of daily between-agent interactions and daily interactions between each agent and the outside community that minimized the mean squared error between the model estimated number of daily positive cases and the daily positive cases at Duke University (Durham, North Carolina) during the Spring 2021 semester. Duke University has used a quantitative polymerase chain reaction–based pooled-testing approach that has been previously described.^1^ After becoming exposed, each agent waited a random number of days before becoming infected. Similarly, after becoming infected, each agent waited a random number of days before becoming recovered. Lognormal distributions, with parameters consistent with current estimates,^21,22,23,24,25,26^ were used to determine these durations. Small changes in disease progression parameter estimates were also made by running simulations using the defaults specified in another agent-based model.^26^ Recovered agents did not become susceptible within the period of the simulations (100 days). To initialize the model, it was assumed each student had a 0.1% chance of being initially exposed and a 0.1% chance of being initially infected. In a student population of 5000, these probabilities yielded an average of 5 infected individuals and 5 exposed individuals on return to campus. All reported simulations were also performed, with each student having a 0.5% chance of being initially exposed and a 0.5% chance of being initially infected, and very similar results were observed under these initial conditions (eFigures 1 and 2 and eTables 1 and 2 in the Supplement).

In addition to the SEIR dynamics, various mitigation strategies were modeled. In simulations with surveillance testing, weekly surveillance tests, with 7 days between tests for each agent and the entire population tested every 7 days, were simulated. For pooled surveillance, agents were grouped into groups of 5 individuals and positive pools were followed by individual tests for each agent in the pool. Pooled and individual tests were assumed to have a uniform delay of 1 day between the test administration and the test result, with a 1.0% false negative rate and a 0.1% false positive rate. On receipt of a positive test result, an agent was moved to isolation for 10 days. In simulations with contact tracing, each positive test led to a random number (possibly 0) of agents being flagged as contacts. Thus, each day, a random number of agents were selected to be reported as contacts of the agents whose positive test results were returned that day. Approximately 15% of the number of reported contacts (or the total number of unisolated, exposed, and infected agents, whichever was smaller) were selected from the current pool of unisolated infected or exposed agents, while the remaining number of reported contacts were selected from among the susceptible or recovered agents. The number of contacts reported per positive test was determined by an empirical distribution based on 2470 contacts reported from 971 positive samples collected by the Duke University contact tracing program during the 2020 to 2021 academic year. The 15% contact tracing effectiveness was estimated using the results of surveillance testing of quarantined contacts at Duke University during the same period. Isolation and quarantine were assumed to be perfect and remove an agent from all interactions. The model architecture used in this study is in eFigure 3 in the Supplement, and model parameters are in eTable 3 in the Supplement.

This work is a simulated modeling study and does not contain economic evaluations of health interventions. Findings are reported in accordance with all relevant Consolidated Health Economic Evaluation Reporting Standards (CHEERS) reporting guidelines. The data used to estimate parameters came from the SARS-CoV-2 surveillance program at Duke University.^1^ This program was reviewed and determined to be exempt by the institutional review board because the surveillance program was determined not to be human participants research.

To assess the effect of various mitigation strategies in an uncertain and potentially evolving environment in Fall 2021, numerical simulations amid several environmental conditions, in combination with several mitigation strategies, were performed. The following modifications to some baseline model parameters were made: the vaccine effectiveness (90%, 75%, and 50%), interaction multiplier (1, 10, and 20), and outside community prevalence (0.1% and 1.0%). Deviations in the vaccine effectiveness reflected uncertainty that was introduced by possible variants and waning immunity. The interaction multipliers accounted for the uncertainty arising in estimates of interaction numbers from increased density on campus and the likelihood of increased numbers of unmasked, indoor interactions inside and outside the classroom compared with the 2020 to 2021 academic year.^13^ A multiplier of M indicates M times as many interactions between agents and with the outside community as estimated during the Spring semester of 2021 when mitigations, such as masking and social distancing, were strictly enforced. Two values of outside community prevalence were simulated to address uncertainty around vaccine uptake levels and potential seasonal effects into the fall.

**Figure 1.**
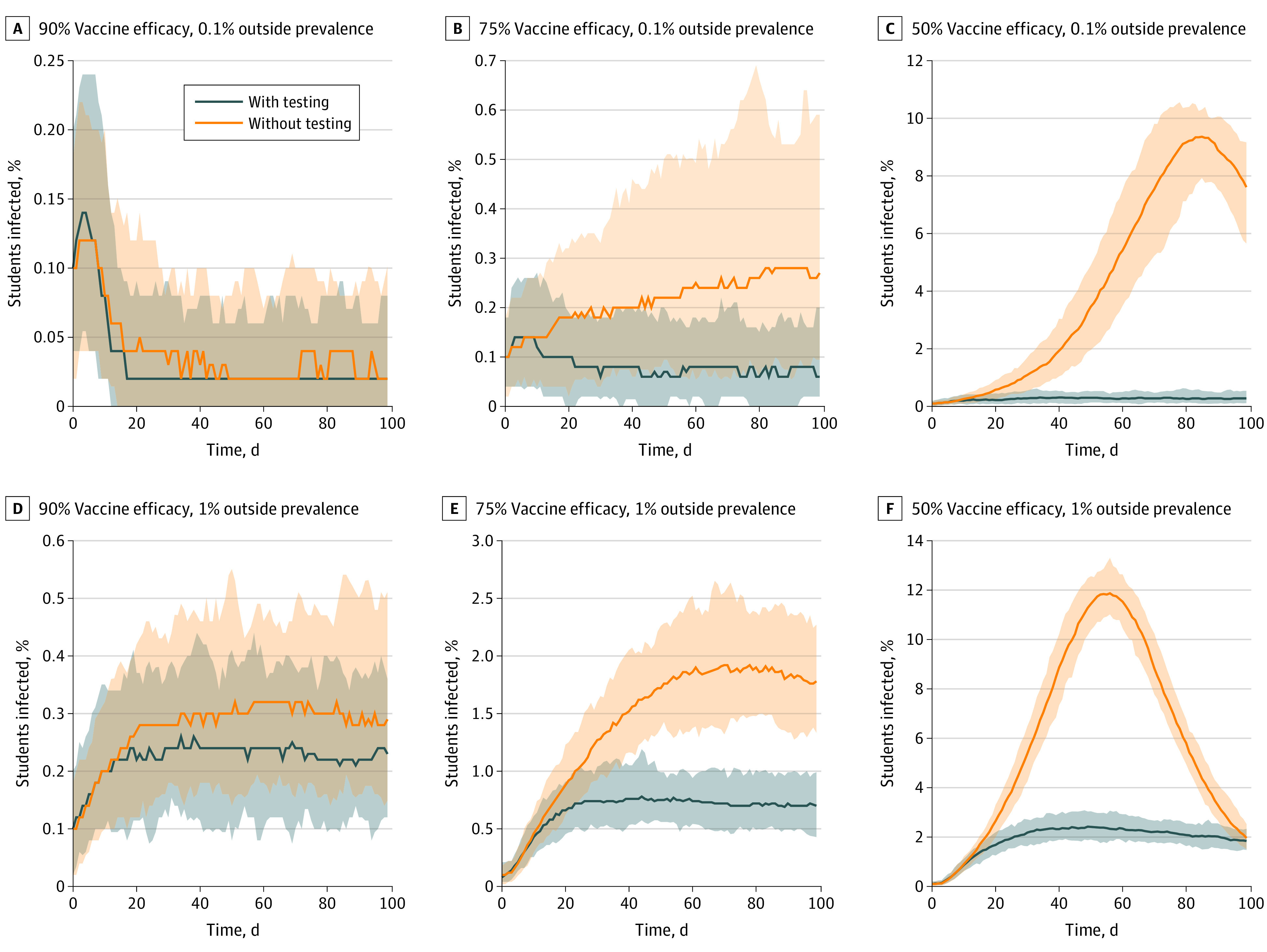
Estimated Daily Infection Prevalence With and Without Surveillance Testing

**Figure 2.**
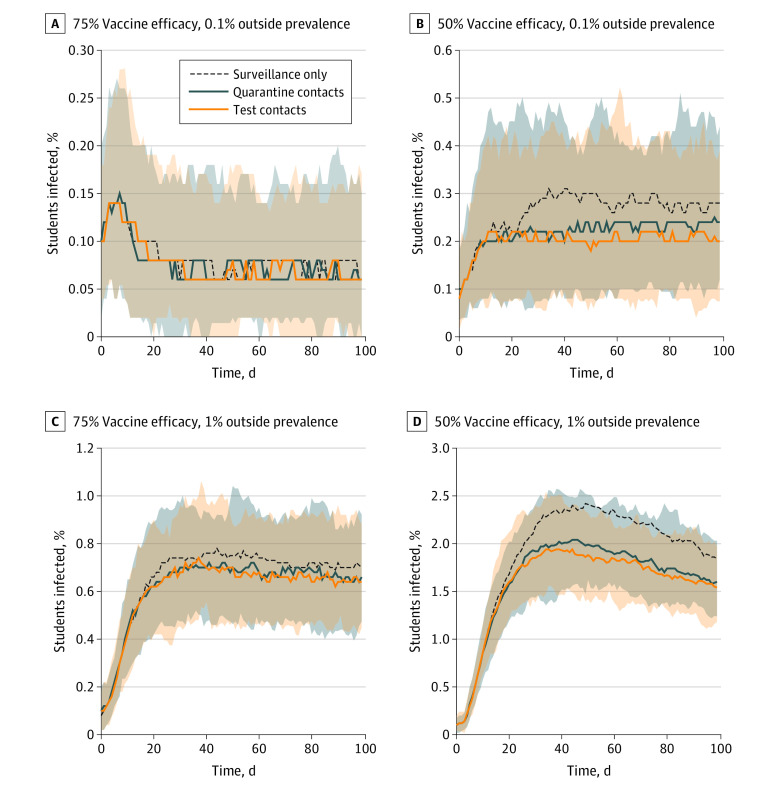
Estimated Daily Infection Prevalence in Quarantining vs Testing Contacts

For each environmental condition, simulations included 1 of the following 4 mitigation strategies: no mitigation, surveillance testing and isolation, and surveillance testing and isolation in combination with contact tracing with either quarantining or targeted testing of reported contacts every 2 days for 10 days. For each choice of environmental condition and mitigation strategy, 100 simulations of randomly initialized populations were performed, with the population seeded with an average of either 0.2% or 1.0% of agents exposed and infected on day 1. Figure 1 and Figure 2 and eFigures 1 and 2 in the Supplement report the medians and 2.75% to 97.5% quantile ranges of daily percentages across the 100 simulations of each model. Table 1 and Table 2 and eTables 1 and 2 in the Supplement report the medians and interquartile ranges of daily percentages across the 100 simulations of each model.

**Table 1.** Estimated Cumulative Infection Prevalence Across Conditions and Mitigations

Population parameters			Mitigation strategies, % (IQR)^a^			
Vaccine effectiveness	Interaction multiplier	Outside community prevalence, %	None	Surveillance only	Surveillance and quarantining contacts	Surveillance and testing contacts
90%	1	0.1	0.2 (0.1)	0.2 (0.1)	0.2 (0.1)	0.2 (0.1)
		1.0	0.4 (0.1)	0.4 (0.1)	0.5 (0.1)	0.4 (0.1)
	10	0.1	0.6 (0.2)	0.5 (0.2)	0.5 (0.1)	0.5 (0.2)
		1.0	3.6 (0.5)	2.9 (0.3)	2.9 (0.3)	2.9 (0.3)
	20	0.1	1.6 (0.5)	0.9 (0.2)	0.9 (0.2)	0.8 (0.2)
		1.0	11.2 (1.3)	6.4 (0.6)	6.1 (0.6)	6.2 (0.5)
75%	1	0.1	0.3 (0.1)	0.3 (0.1)	0.3 (0.1)	0.3 (0.1)
		1.0	0.8 (0.2)	0.8 (0.2)	0.8 (0.2)	0.8 (0.2)
	10	0.1	2.8 (1.1)	1.2 (0.3)	1.1 (0.3)	1.1 (0.2)
		1.0	18.5 (2.0)	8.6 (0.6)	8.1 (0.7)	8.0 (0.7)
	20	0.1	55.8 (6.1)	3.6 (1.0)	3.0 (0.8)	2.7 (0.6)
		1.0	74.9 (1.4)	24.5 (1.8)	21.1 (1.5)	20.1 (1.3)
50%	1	0.1	0.4 (0.1)	0.4 (0.1)	0.3 (0.1)	0.3 (0.1)
		1.0	1.6 (0.2)	1.5 (0.2)	1.5 (0.2)	1.5 (0.3)
	10	0.1	55.8 (6.0)	3.6 (1.0)	3.0 (0.8)	2.7 (0.6)
		1.0	75.0 (1.4)	24.5 (1.6)	21.2 (1.5)	20.2 (1.1)
	20	0.1	96.6 (0.4)	44.8 (7.3)	26.5 (5.7)	20.1 (5.1)
		1.0	97.4 (0.3)	74.3 (2.0)	61.7 (2.2)	57.6 (2.1)

^a^ The medians (IQRs) of the fraction of cumulative infections in a modeled population of 5000 individuals over 100 simulations of each choice of population parameters and mitigation strategy. For mitigation strategies involving contact tracing, the contact tracing efficacy was fixed at 15%. Simulations were initialized with 0.1% (5) expected initial exposures and 0.1% (5) expected initial infections.

**Table 2.** Estimated Maximum Fraction of Isolated and Quarantined Individuals

Population parameters			Mitigation strategies % (IQR)^a^		
Vaccine effectiveness	Interaction multiplier	Outside community prevalence, %	Surveillance only	Surveillance and quarantining contacts	Surveillance and testing contacts
90%	1	0.1	0.1 (0.1)	0.4 (0.2)	0.1 (0.1)
		1.0	0.1 (0.1)	0.5 (0.2)	0.1 (0.1)
	10	0.1	0.2 (0.1)	0.5 (0.2)	0.2 (0.1)
		1.0	0.4 (0.1)	1.3 (0.2)	0.5 (0.1)
	20	0.1	0.2 (0.1)	0.6 (0.2)	0.2 (0.1)
		1.0	0.9 (0.1)	2.5 (0.4)	0.9 (0.1)
75%	1	0.1	0.1 (0.1)	0.4 (0.2)	0.1 (0.1)
		1.0	0.2 (0.1)	0.5 (0.2)	0.2 (0.1)
	10	0.1	0.2 (0.1)	0.7 (0.3)	0.2 (0.1)
		1.0	1.1 (0.2)	3.3 (0.6)	1.1 (0.2)
	20	0.1	0.6 (0.2)	1.5 (0.5)	0.5 (0.1)
		1.0	3.2 (0.4)	7.7 (0.9)	2.7 (0.3)
50%	1	0.1	0.2 (0.1)	0.4 (0.2)	0.1 (0.1)
		1.0	0.3 (0.1)	0.8 (0.2)	0.3 (0.0)
	10	0.1	0.6 (0.2)	1.5 (0.5)	0.5 (0.1)
		1.0	3.2 (0.4)	7.8 (1.0)	2.7 (0.3)
	20	0.1	7.8 (1.4)	12.0 (3.2)	3.3 (0.8)
		1.0	14.7 (1.2)	28.2 (2.8)	.3 (1.0)

^a^ The medians (IQRs) of the daily maximum fraction of agents in isolation or quarantine in a modeled population of 5000 individuals for 100 simulations of each choice of population parameters and mitigation strategies. For mitigation strategies involving contact tracing, the contact tracing efficacy was fixed at 15%. Simulations were initialized with 0.1% (5) expected initial exposures and 0.1% (5) expected initial infections.

## Results

Regardless of prevalence levels in the off-campus community, at 90% vaccine effectiveness, weekly surveillance testing was associated with only a modest reduction in campus infections, in most conditions reducing the median cumulative infections by less than 1 percentage point (Figure 1 and Table 1). A reduction in median cumulative prevalence by approximately 5 percentage points was estimated at 90% vaccine effectiveness only in the most extreme case of an increase in daily interactions by a factor of 20 and 1% outside community prevalence (Figure 1 and Table 1).

Infection dynamics appeared different in conditions in which vaccine effectiveness dipped to 75% or 50%. At 75% vaccine effectiveness and a daily interactions multiplier of 10, weekly surveillance testing was associated with a rapid drop of infections to a baseline, whereas in the no testing scenario, infection numbers rose slowly during the semester (Figure 1). In this scenario, surveillance testing was associated with a reduction in the number of infections by 53% to 62% vs a no testing regime (Figure 1; Table 1; eTable 1 in the Supplement). If vaccine effectiveness dropped to 50%, then weekly surveillance testing was even more important for limiting the spread of infections. With a daily interactions multiplier of 10, surveillance testing was associated with a substantial reduction in cumulative infections and peak daily infections, whereas in the absence of testing, daily infections peaked between 8% and 12% of the population and then dropped toward baseline levels as most of the campus population became protected from reinfection for the duration of the semester (Figure 1 and Table 1).

Does contact tracing and quarantine help limit viral transmission in the context of weekly surveillance testing, and how does testing reported contacts with increased frequency compare with quarantining contacts? The medians and ranges of model predictions involving contact tracing are shown in Figure 2 and eFigure 2 in the Supplement. Model simulations estimated that quarantine does not substantially reduce infection numbers at 90% vaccine effectiveness and only marginally reduces infections over the course of the semester at 75% vaccine effectiveness (Figure 2; eFigure 2 in the Supplement; Table 1; eTable 1 in the Supplement). At most, quarantine was estimated to reduce infection totals by 16% to 17%, assuming a vaccine effectiveness of 50% and an interaction multiplier of 20. In this scenario, testing every 2 days was a more effective strategy than quarantining reported contacts. Across the conditions, increased testing of contacts appeared to be at least as effective as quarantine in limiting the spread of infections (Table 1; eTable 1 in the Supplement).

For planning purposes, it is important for universities to estimate the number of rooms needed for quarantine and isolation. The maximum number of daily isolations and quarantines were estimated as a percentage of the total population, assuming 10-day isolation and quarantine durations. With surveillance testing and quarantine, the estimated median maximum number in isolation or quarantine ranged between 20 for a population of 5000 (0.4%) and 1410 (28.2%) (Table 2). For the 90% effectiveness scenario, the maximum isolated and quarantined are likely needed at the very beginning of the semester during which the peak of infections occur, and for the 75% and 50% scenarios, the peak of infections occurred more toward the middle or end of the semester (Figure 1).

As an alternative to quarantine, increased testing of contact-traced students (every 2 days) was simulated. Model outputs indicated that increased testing is associated with about the same number of infections as quarantine, suggesting that this increased testing cadence may be a viable alternative to quarantine on campuses with 100% of students vaccinated. An estimated benefit of increased testing over quarantine is a substantial reduction in the maximum and total number of students in isolation and quarantine (Table 2; eTable 2 in the Supplement), thus conserving considerable space and human resources needed for quarantine while allowing students to continue to fully engage in the campus learning environment.

## Discussion

There is a growing need to reevaluate the importance of university COVID-19 protocols given the availability of effective vaccines. Most universities are encouraging vaccination, and many have mandated vaccination for all students. Recent guidance from the CDC suggests that mitigations, such as testing, quarantine, and masking, are not necessary for vaccinated populations,^13^ and universities are likely to adopt these guidelines as university policy. Relaxing mitigations will necessarily increase the number of interactions within student populations; thus, without surveillance testing, limiting infections on campus will mostly rely on the capability of vaccines to prevent infection and transmission.

Starting with the assumption that 100% of the student population will be vaccinated, if vaccines reduce the probability of transmission by 90%, total infections are estimated to be low, and even a surge of infections quickly decays in the absence of surveillance testing (Figure 1 and Table 1). However, if vaccine effectiveness drops to 75%, infection dynamics change, and many on-campus infections can be observed in the absence of testing. At 50% vaccine effectiveness, the model predicts the possibility of on-campus community spread and exponential growth of infections that could cause substantial outbreaks. At 75% and 50% effectiveness, surveillance testing alone is associated with a marked reduction in infections (Figure 1; eFigure 1 in the Supplement; Table 1; eTable 1 in the Supplement). These results are qualitatively consistent with previous modeling work pertaining to university populations in the Fall 2021 semester.^27^ Indeed, under somewhat different models and parameters than those considered in this article, they also found that “less effective vaccines or incidence of new variants may require additional intervention such as screening testing” for universities to reopen safely.^27^

Vaccine effectiveness could be reduced to lower levels by multiple mechanisms. First, while the BioNTech 162b2 and messenger RNA-1273 vaccines are reported to be 90% efficient at blocking infection and transmission,^2,3,4^ the Ad26.COV2.S vaccine efficacy is considerably lower at 67%.^5^ Thus, many individuals with the Ad26.COV2.S vaccine on campus could push the average effectiveness less than 75%. Second, it is not yet known how long vaccine-induced immunity will last or remain at high levels. Finally, several genetic variants exhibit some resistance to vaccine-induced neutralizing antibodies,^6,7,8,9^ and while current vaccines are reported to be effective against current variants, they may not have maximum effectiveness against current variants.^28,29^ Furthermore, as populations have become partially vaccinated, there is selective pressure favoring variants that can bypass immunity.

In addition to vaccine effectiveness, other factors also are associated with on-campus infection rates. Interactions may increase as mitigations such as masking are relaxed, and increased interactions are associated with higher infection rates at all vaccine efficiencies (Table 1; eTable 1 in the Supplement). However, it is hard to predict how much interactions may increase, so it is not clear how relevant the choices of interaction levels are. Increased off-campus prevalence is also associated with an increase in campus infection rates. Off-campus prevalence rates are difficult to predict and will likely depend on the percentage of the off-campus population that is vaccinated. Thus, universities may be differentially affected based on local vaccination uptake rates.

In the context of weekly surveillance testing, the role of quarantine was also explored (Figure 2; eFigure 2 in the Supplement). The results indicated that quarantine is associated with only a modest reduction in infection rates in most scenarios unless vaccine effectiveness drops to 50% (Figure 2 and Table 1). Model simulations that replaced quarantine with an increased testing frequency were estimated to be as efficient as quarantine at limiting infections. These findings support the suggestion that quarantine may be unnecessary for a well-vaccinated student population.

### Limitations

Modeling approaches such as the one used here have various limitations. The accuracy of self-reporting of contacts is unknown, may depend on disease prevalence, and may change in the future. However, the conclusions of this study were qualitatively insensitive to changes in the efficacy of contact tracing (not reported). Moreover, in the absence of information on interaction networks, the model assumed homogeneous mixing. Finally, the model only tracked infection dynamics and did not account for the association of vaccines with disease severity. If disease severity is diminished to acceptable levels in vaccinated individuals,^3,4^ the tolerance for infections on campus may be increased.

Although it is expected that these findings will be generally applicable to universities, important parameters in the model were fit to empirical data collected at Duke University during the 2020 to 2021 academic year; therefore, outcomes may be context specific. Also, schools with large student populations or limited resources may be unable to implement weekly surveillance testing.

A further limitation of this study stems from uncertainty about the association vaccines will have with the course of infection. Rather than modify disease progression parameters for a vaccinated population, the model described in this article directly modified transmission probabilities so that a vaccine effectiveness of X% was expected to prevent that percentage of potential exposures. This is not the only possible model choice that could be used to explain observed vaccine efficacies, but it is consistent with emerging evidence that vaccinated individuals can become infected.^10,30,31^ Furthermore, this study only modeled a fully vaccinated student population and so only directly models universities with a vaccine mandate, although the number of infections would only be greater in partially vaccinated populations. It is not yet clear whether infected individuals who are vaccinated will systematically have lower viral loads and thus be less likely to transmit.^32,33^ If true, then infection rates may be lower than what the model predicted.

Aside from limiting viral transmission on campus, surveillance testing provides other useful public health information. Sequencing of surveillance positives has been used to track the arrival or expansion of variants that have attributes, such as increased transmissibility or disease severity and enhanced resistance to neutralizing antibodies. The constitution of variants in the population could inform policies on mitigations. Moreover, as policies on gatherings, masking, and other mitigations are relaxed, surveillance testing provides real-time feedback on whether relaxations are triggering unwanted spread of SARS-CoV-2 so that timely adjustments can be made. Finally, it is not yet clear how long vaccine-induced immunity will last in the face of new variants, and continued surveillance testing is likely to reveal signatures that are associated with waning immunity.

## Conclusions

This simulated modeling study of infection dynamics on a college campus in which 100% of the student body was vaccinated suggested that surveillance testing had an important role in limiting infections in all but the most optimistic conditions, which may be unrealistic in the face of potentially decreasing vaccine effectiveness because of variants or waning immunity. Simulations involving surveillance testing suggest that quarantine is associated with only a modest reduction in infections in most scenarios and may be replaced by increased testing of reported contacts.

## Notes

Supplement. eFigure 1. Estimated daily infection prevalences, with and without surveillance testing (pessimistic initial infections and exposures) eFigure 2. Estimated daily infection prevalences, quarantining vs. testing contacts (pessimistic initial infections and exposures) eFigure 3. Overview of model architecture eTable 1. Estimated cumulative infection prevalences across conditions and mitigations (pessimistic initial infections and exposures) eTable 2. Estimated maximum fraction of isolated and quarantined individuals (pessimistic initial infections and exposures) eTable 3. Model parameters
