# Supplementary material for: Assessment of Simulated Surveillance Testing and Quarantine in a SARS-CoV-2–Vaccinated Population of Students on a University Campus
Source: JAMA Health Forum. 2021 Oct 1;2(10):e213035. doi: 10.1001/jamahealthforum.2021.3035 (PMC8727034; doi:10.1001/jamahealthforum.2021.3035)
Supplement: Supplement. — eFigure 1. Estimated daily infection prevalences, with and without surveillance testing (pessimistic initial infections and exposures) eFigure 2. Estimated daily infection prevalences, quarantining vs. testing contacts (pessimistic initial infections and exposures) eFigure 3. Overview of model architecture eTable 1. Estimated cumulative infection prevalences across conditions and mitigations (pessimistic initial infections and exposures) eTable 2. Estimated maximum fraction of isolated and quarantined individuals (pessimistic initial infections and exposures) eTable 3. Model parameters [file jamahealthforum-e213035-s001.pdf]

Supplemental Online Content

Motta FC, McGoff KA, Deckard A, et al. Assessment of simulated surveillance testing and quarantine in a SARS-CoV-2–vaccinated population of students on a university campus. *JAMA Health Forum*. 2(10):e213035. doi:10.1001/jamahealthforum.2021.3035

eFigure 1. Estimated daily infection prevalences, with and without surveillance testing (pessimistic initial infections and exposures)
eFigure 2. Estimated daily infection prevalences, quarantining vs. testing contacts (pessimistic initial infections and exposures)
eFigure 3. Overview of model architecture
eTable 1. Estimated cumulative infection prevalences across conditions and mitigations (pessimistic initial infections and exposures)
eTable 2. Estimated maximum fraction of isolated and quarantined individuals (pessimistic initial infections and exposures)
eTable 3. Model parameters

This supplemental material has been provided by the authors to give readers additional information about their work.

© 2021 Motta FC et al. *JAMA Health Forum.*

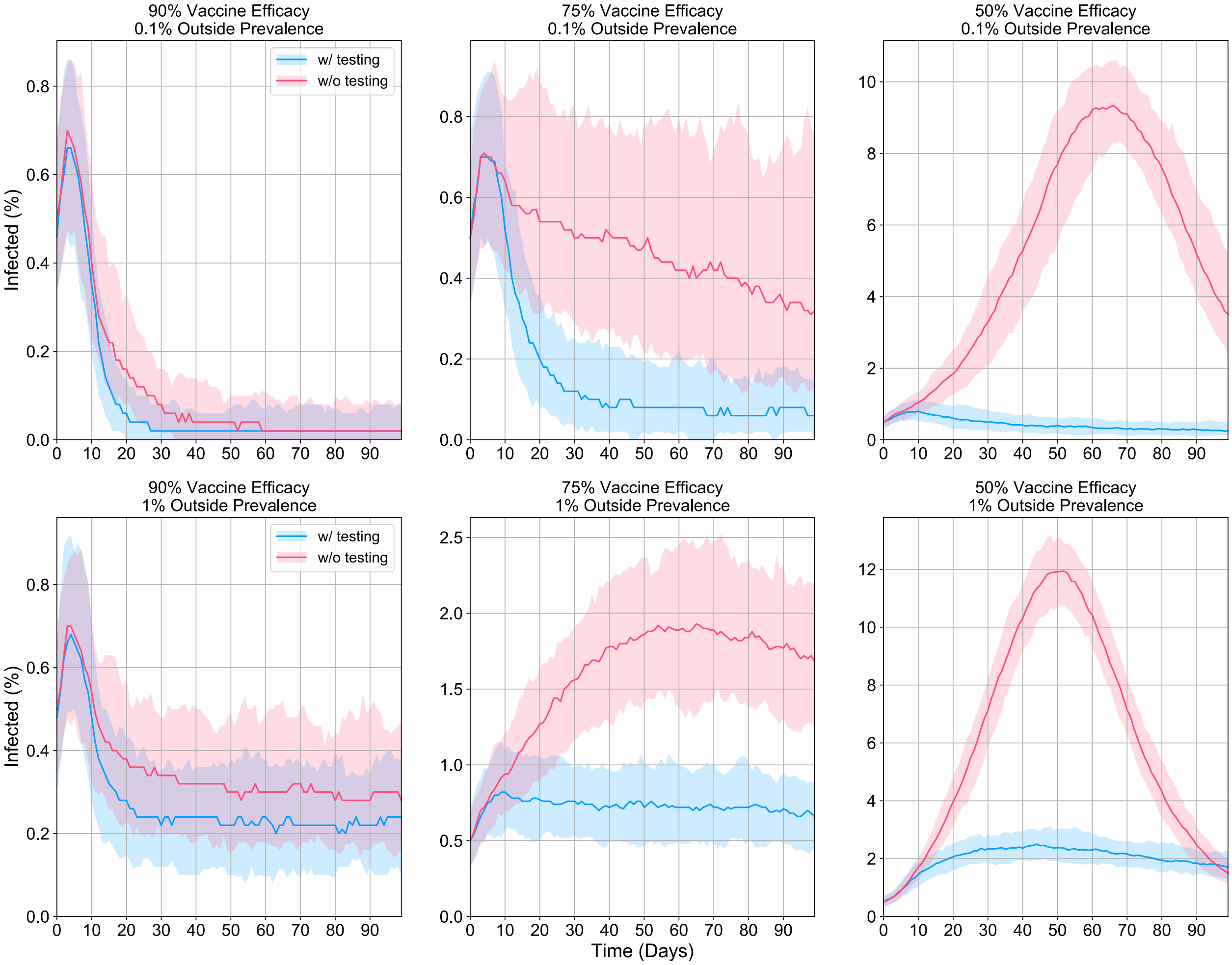

eFigure 1. Estimated daily infection prevalence, with and without surveillance testing. The medians (dark lines) and 2.75%-97.5% quantile ranges (shaded bands) of the daily infection prevalence in the modelled population of 5000 individuals over 100 simulations assuming an interaction multiplier of 10. The outside community infection prevalence was fixed at 0.1% (top) or 1.0% (bottom), with vaccine effectiveness chosen to be 90% (left), 75% (middle) or 50% (right). Simulations were initialized with 0.5% (25) expected initial exposures and 0.5% (25) expected initial infections. All other model parameters were identical across simulations.

© 2021 Motta FC et al. *JAMA Health Forum.*

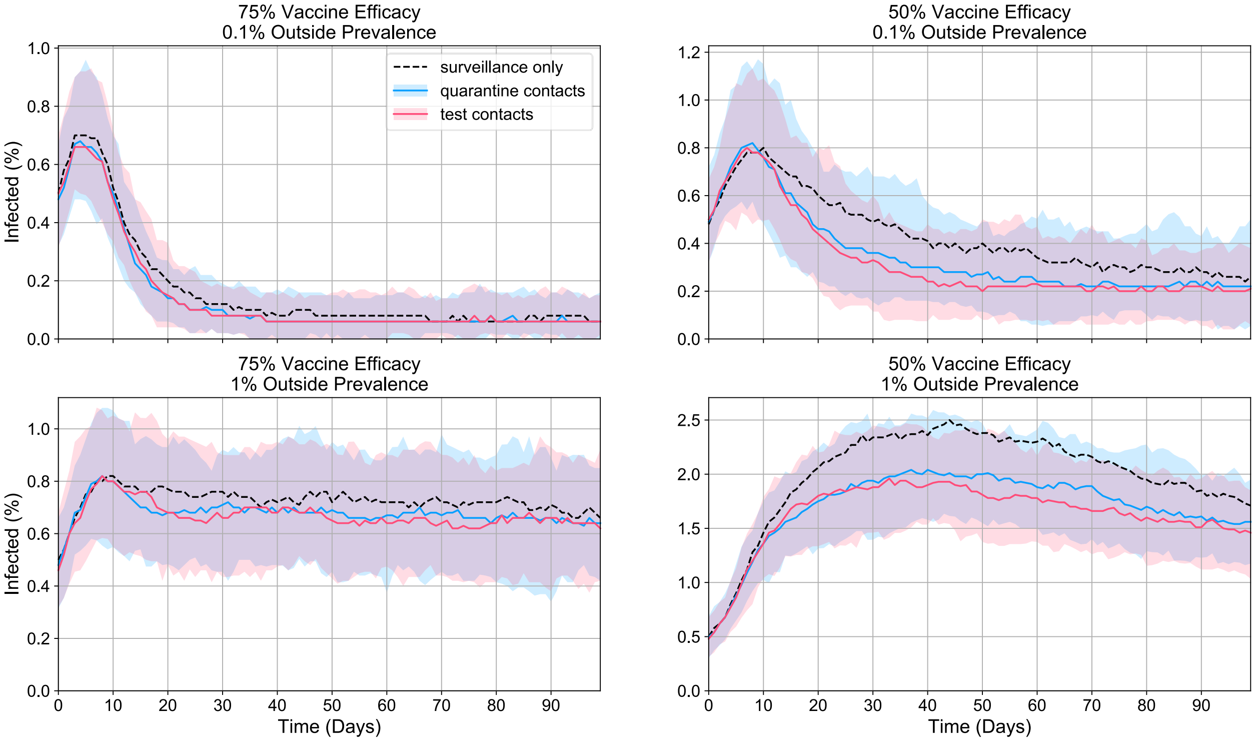

eFigure 2. Estimated daily infection prevalence, quarantining vs. testing contacts. The medians (dark lines) and 2.75%-97.5% quantile ranges (shaded bands) of the daily infection prevalence in a modelled population of 5000 individuals over 100 simulations assuming an interaction multiplier of 10 and either surveillance testing only, surveillance testing with quarantining of contacts, or surveillance testing with testing of contacts every 2 days. The outside community infection prevalence was fixed at 0.1% (top) or 1.0% (bottom), with vaccine effectiveness chosen to be 75% (left) or 50% (right). Contact tracing efficacy was fixed at 15%. Simulations were initialized with 0.5% (25) expected initial exposures and 0.5% (25) expected initial infections. All other model parameters were identical across simulations.

© 2021 Motta FC et al. *JAMA Health Forum.*

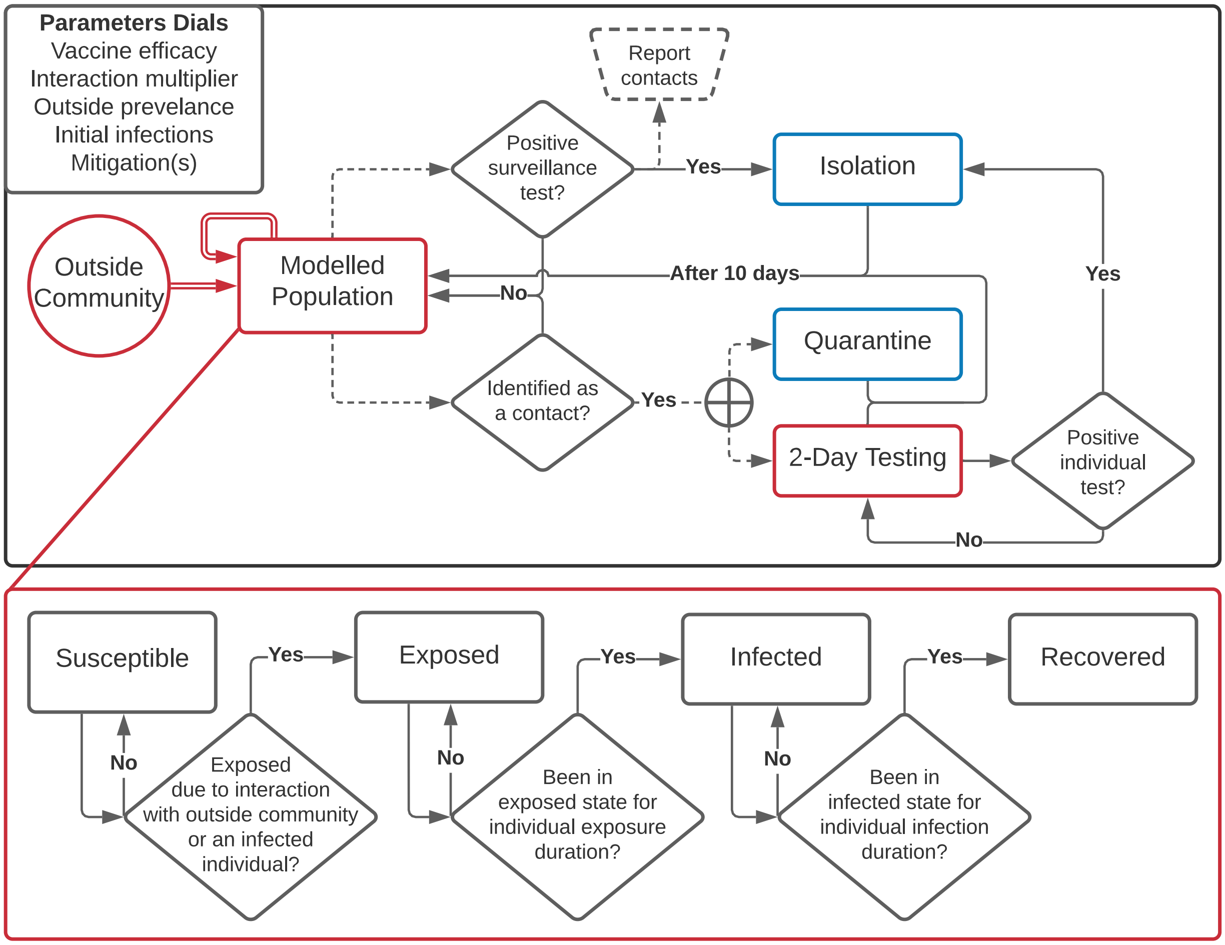

eFigure 3. Overview of model architecture. (Top) Flowchart describing each of the four mitigation strategies considered in this study: 1) no testing, 2) surveillance testing only, 3) surveillance with quarantining of contacts, and 4) surveillance with follow-up testing of contacts every 2 days. Dashed arrows indicate optional pathways if the specified mitigation is being modelled. Individuals in red boxes may have interactions with others in the modelled population, while individuals with blue attributes are isolated from the modelled population and outside community. (Bottom) Flowchart of SEIR model and agent health state progression. Arrows indicate transitions between states and daily update rules.

© 2021 Motta FC et al. *JAMA Health Forum.*

Population Parameters			Mitigation Strategies, % (IQR)			
Vaccine Effectiveness	Interaction Multiplier	Outside Community Prevalence	None	Surveillance Only	Surveillance and Quarantining Contacts	Surveillance and Testing Contacts
90%	1	0.1%	1.0% ± 0.2%	1.0% ± 0.2%	1.0% ± 0.2%	1.0% ± 0.2%
		1.0%	1.3% ± 0.2%	1.2% ± 0.2%	1.2% ± 0.2%	1.2% ± 0.2%
	10	0.1%	1.7% ± 0.3%	1.4% ± 0.2%	1.4% ± 0.3%	1.4% ± 0.2%
		1.0%	4.8% ± 0.6%	3.8% ± 0.4%	3.8% ± 0.4%	3.8% ± 0.3%
	20	0.1%	3.7% ± 0.9%	1.9% ± 0.4%	1.9% ± 0.3%	1.9% ± 0.2%
		1.0%	13.0% ± 1.4%	7.4% ± 0.7%	7.0% ± 0.5%	7.1% ± 0.8%
75%	1	0.1%	1.1% ± 0.2%	1.1% ± 0.2%	1.1% ± 0.2%	1.1% ± 0.2%
		1.0%	1.7% ± 0.3%	1.6% ± 0.3%	1.6% ± 0.3%	1.6% ± 0.2%
	10	0.1%	6.3% ± 1.4%	2.4% ± 0.5%	2.1% ± 0.4%	2.1% ± 0.3%
		1.0%	20.7% ± 2.0%	9.6% ± 0.7%	9.0% ± 0.8%	8.8% ± 0.8%
	20	0.1%	66.5% ± 2.6%	5.8% ± 1.0%	4.7% ± 1.0%	4.5% ± 0.8%
		1.0%	75.7% ± 1.4%	26.1% ± 1.9%	22.1% ± 1.5%	21.2% ± 1.5%
50%	1	0.1%	1.3% ± 0.2%	1.2% ± 0.2%	1.2% ± 0.2%	1.2% ± 0.2%
		1.0%	2.6% ± 0.3%	2.3% ± 0.4%	2.3% ± 0.3%	2.3% ± 0.3%
	10	0.1%	66.5% ± 2.7%	5.8% ± 1.0%	4.7% ± 1.0%	4.5% ± 0.8%
		1.0%	75.8% ± 1.4%	25.7% ± 2.0%	22.0% ± 1.5%	21.2% ± 1.4%
	20	0.1%	96.7% ± 0.4%	54.3% ± 5.6%	33.7% ± 6.0%	25.4% ± 5.1%
		1.0%	97.5% ± 0.4%	74.3% ± 1.5%	62.4% ± 2.5%	58.7% ± 2.3%

eTable 1. Estimated cumulative infection prevalence across conditions and mitigations. The medians and interquartile ranges of the fraction of cumulative infections in a modelled population of 5000 individuals over 100 simulations of each choice of population parameters and mitigation strategy. For mitigation strategies involving contact tracing, the contact tracing efficacy was fixed at 15%. Simulations were initialized with 0.5% (25) expected initial exposures and 0.5% (25) expected initial infections.

© 2021 Motta FC et al. *JAMA Health Forum.*

Population Parameters			Mitigation Strategies, % (IQR)		
Vaccine Effectiveness	Interaction Multiplier	Outside Community Prevalence	Surveillance Only	Surveillance and Quarantining Contacts	Surveillance and Testing Contacts
90%	1	0.1%	0.7% ± 0.1%	2.0% ± 0.6%	0.7% ± 0.1%
		1.0%	0.7% ± 0.2%	2.1% ± 0.7%	0.7% ± 0.2%
	10	0.1%	0.7% ± 0.2%	2.1% ± 0.7%	0.8% ± 0.2%
		1.0%	0.8% ± 0.2%	2.4% ± 0.6%	0.9% ± 0.2%
	20	0.1%	0.8% ± 0.2%	2.2% ± 0.7%	0.9% ± 0.2%
		1.0%	1.0% ± 0.1%	2.9% ± 0.6%	1.0% ± 0.2%
75%	1	0.1%	0.7% ± 0.1%	2.0% ± 0.5%	0.7% ± 0.2%
		1.0%	0.7% ± 0.2%	2.1% ± 0.7%	0.8% ± 0.2%
	10	0.1%	0.9% ± 0.2%	2.4% ± 0.7%	0.9% ± 0.2%
		1.0%	1.2% ± 0.1%	3.4% ± 0.5%	1.2% ± 0.1%
	20	0.1%	1.0% ± 0.2%	3.1% ± 0.9%	1.1% ± 0.3%
		1.0%	3.3% ± 0.4%	7.8% ± 0.9%	2.7% ± 0.3%
50%	1	0.1%	0.7% ± 0.2%	2.0% ± 0.5%	0.8% ± 0.2%
		1.0%	0.7% ± 0.2%	2.1% ± 0.6%	0.8% ± 0.2%
	10	0.1%	1.0% ± 0.2%	3.1% ± 0.9%	1.1% ± 0.3%
		1.0%	3.1% ± 0.4%	7.7% ± 1.0%	2.7% ± 0.3%
	20	0.1%	8.2% ± 1.4%	12.9% ± 2.5%	3.4% ± 0.9%
		1.0%	14.5% ± 1.0%	28.2% ± 2.6%	10.2% ± 1.0%

eTable 2. Estimated maximum fraction of isolated and quarantined individuals. The medians and interquartile ranges of the daily maximum fraction of agents in isolation or quarantine in a modelled population of 5000 individuals for 100 simulations of each choice of population parameters and mitigation strategies. For mitigation strategies involving contact tracing, the contact tracing efficacy was fixed at 15%. Simulations were initialized with 0.5% (25) expected initial exposures and 0.5% (25) expected initial infections.

© 2021 Motta FC et al. *JAMA Health Forum.*

Variable	Description	Value
**Disease Progression**		
Duration of exposed	The number of days agents stay in the exposed state.	(*)
Duration of infected	The number of days agents stay in the infected state.	(**)
**Population**		
Size	The number of individuals in the modelled population.	5000
Initial exposed	The average fraction of individuals initially in the exposed state.	0.1% / 0.5%
Initial infected	The average fraction of individuals initially in the infected state.	0.1% / 0.5%
Baseline daily interactions	The baseline number of unique daily interactions between members of the modelled population on average.	1.67 (†)
Baseline interaction exposure probability	The baseline probability that a susceptible-infected interaction will result in an exposure.	2.60%
Baseline outside daily interactions	The baseline number of unique daily interactions individuals will have with the outside community on average.	0.995 (†)
Baseline outside interaction exposure probability	The baseline probability that an interaction between a susceptible member of the modelled population and the outside community will result in an exposure.	2.60%
Outside prevalence	The current fraction that is infected in the outside community.	0.1% / 1.0%
Interaction multiplier	Factor by which the baseline number of interactions is multiplied.	1 / 10 / 20
Vaccine effectiveness	Percent reduction in the baseline interaction exposure probability.	90% / 75% / 50%
**Surveillance and Individual Testing**		
Days between surveillance tests	The number of days between tests of individual agents, and the number of days required to test the entire population exactly once.	7
Testing pool size	The number of tests pooled together for initial screening.	5
Test FPR	The false positive rate of pooled and individual tests.	0.1%
Test FNR	The false negative rate of pooled and individual tests.	1.0%
Test results delay	The number of days until test results become available.	1
Isolation duration	The number of days a positive case spends in isolation.	10
**Contact Tracing Parameters**		
Contacts reported	The number of contacts an agent will report if they test positive	(***)
Tracing effectiveness	The maximum fraction of reported contacts who are exposed or infected at the time of the contact trace.	15%
Quarantine duration	The number of days a reported contact of a positive test spends in quarantine (or undergoes additional testing)	10

eTable 3. Model parameters. The set of model parameters used in this study. Disease progression parameters and reported contacts determined by parameterized distributions: (*) lognormal(mean=4.6, stdev=4.8, minimum=3) or lognormal(mean=4.5, stdev=1.5, minimum=1), (**) 1.1% drawn from lognormal(mean=14.0, stdev=2.4, minimum=1) and 98.9% drawn from lognormal(mean=8.0, stdev=2.0, minimum=1) or lognormal(mean=8.0, stdev=2.0, minimum=1), and (***) the empirical distribution from the Duke University 2020-21 academic year with an average of 2.54 contacts per positive test. The function lognormal(mean, stdev, minimum) returns a sample of a log normal distribution, with specified mean and standard deviation, and with values rounded to the nearest integer, with values less than the specified minimum replaced by that minimum. (†) The parameters determining daily interactions and outside interactions were found, among 500 samples, to minimize the total squared error between the model estimated daily positive cases (averaged over 100 simulations) and the measured positive cases at Duke University during the Spring 2021 semester. The 500 samples were drawn from the two-dimensional parameter space [0,20]x[0,20] using Bayesian optimization starting with the uniform prior.

© 2021 Motta FC et al. *JAMA Health Forum.*
